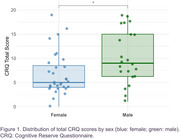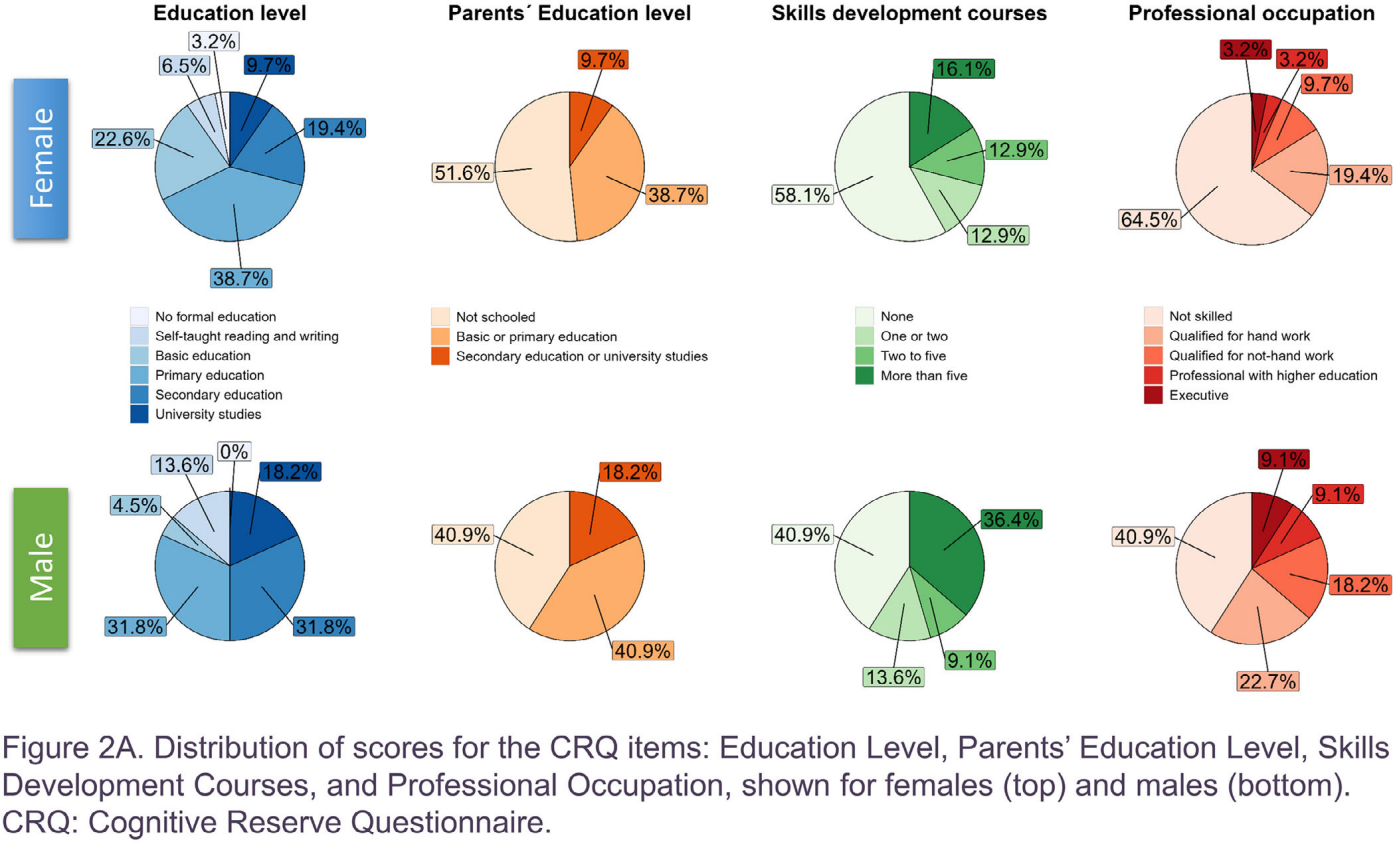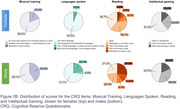# Sex Differences in Cognitive Reserve: Findings from a Brief Questionnaire in Biologically Confirmed Alzheimer's Disease

**DOI:** 10.1002/alz70858_106927

**Published:** 2025-12-25

**Authors:** Marta Rodríguez‐Camacho, María del Mar Martínez‐Salmerón, Luis Andrade‐Zumárraga, Estefanía Drzewiscki, David Romera‐Morales, Consuelo Barranco Criado, Almudena Aguilera del Moral, Firma Rodríguez Sánchez, María del Mar Iglesias‐Espinosa, José Enrique Arriola‐Infante

**Affiliations:** ^1^ Hospital Universitario Torrecárdenas, Almería, Almería, Spain; ^2^ Hospital Universitario de Ceuta, Ceuta, Ceuta, Spain

## Abstract

**Background:**

Cognitive reserve is an increasingly studied concept. According to this framework, individuals with higher reserve can better cope with pathology, delaying symptom onset. The Cognitive Reserve Questionnaire (CRQ) is a brief (<2 min) eight‐item scale validated in the Spanish population. Recently, it has been used to create normative data for a screening cognitive test (TMA‐93), showing greater variability in scores than the isolated education level. This study aimed to explore sex differences in cognitive reserve, as measured by the CRQ, in a biologically‐confirmed Alzheimer's disease (AD) cohort enriched with illiterate individuals from southern Spain.

**Method:**

Cross‐sectional study, including patients from the Outpatient Memory Clinic at Torrecárdenas Hospital in Almería (2023–2024), diagnosed with AD based on CSF biomarkers (*p*‐tau 181/Aβ42 < 0.023; Elecsys automated platform). Baseline sociodemographic data (age, sex, education, Fototest score) and biomarker levels (Aβ42, *p*‐tau 181, t‐tau) were collected, along with CRQ scores, reported by a reliable informant. We analyzed sex differences in total CRQ scores (Mann‐Whitney U test) and examined the distribution of each CRQ item score across sexes.

**Result:**

53 AD patients (median age: 70 years, IQR: 66–75) were included (31 females, 22 males). No significant differences were found between sexes in age, education level, Fototest score, or biomarker levels (Table). Notably, only 37.7% of participants had education beyond primary school. Both sexes exhibited low cognitive reserve (median CRQ score: 7/25, IQR: 4–11), with significantly lower scores in females (Figure 1; W = 212; *p* = 0.01977). At the item level, males scored higher across all items (Figure 2A, 2B), with notable differences in occupation, reading, skills development courses, and languages spoken.

**Conclusion:**

Cognitive reserve, which moderates the relationship between brain pathology and cognitive outcomes, may be influenced by sex. Developing feasible cognitive reserve assessments applicable to illiterate individuals, which also account for sex differences, is essential. Women may have lower cognitive reserve, particularly in populations with a high prevalence of illiteracy. Future studies are needed to explore the sex‐specific moderating effects of AD pathology on cognition and the potential generational changes in cognitive reserve across sexes.